# Identification of a novel protein truncating mutation p.Asp98* in *XPC* associated with xeroderma pigmentosum in a consanguineous Pakistani family

**DOI:** 10.1002/mgg3.1060

**Published:** 2020-01-10

**Authors:** Muhammad Z. Ali, Jasmin Blatterer, Muzammil A. Khan, Erich Schaflinger, Erwin Petek, Safeer Ahmad, Ejazullah Khan, Christian Windpassinger

**Affiliations:** ^1^ Gomal Centre of Biochemistry and Biotechnology Gomal University Dera Ismail Khan Khyber Pakhtunkhwa Pakistan; ^2^ Diagnostic & Research Institute of Human Genetics Medical University of Graz Graz Austria

**Keywords:** frameshift mutation, homozygosity mapping, Pakistani family, xeroderma pigmentosum, *XPC*

## Abstract

**Background:**

Xeroderma pigmentosum (XP) is a rare genetic disorder, which is characterized by hyper‐sensitivity to solar ultraviolet (UV) radiation. Clinical consequences of sun exposure are skin lesions and an increased risk of developing skin cancer. Genetic studies have identified eight genes associated with xeroderma pigmentosum. The proteins encoded by these genes are mainly involved in DNA repair mechanisms.

**Methods:**

Molecular genetic characterization of patients with xeroderma pigmentosum involved positional cloning methods such as homozygosity mapping and subsequent candidate gene analysis. Mutation screening was performed through Sanger DNA sequencing.

**Results and Discussion:**

In this case study, we report a novel protein truncating mutation in *XPC* associated with autosomal recessive xeroderma pigmentosum in a consanguineous Pakistani family. Genetic mapping revealed a novel single base insertion of a thymine nucleotide NM_004628.4: c.291dupT (c.291_292insT) in the second exon of *XPC*. The identified mutation leads to a premature stop codon (TGA) at amino acid position 98 (p.Asp98*) and thus presumably results in a truncated protein. The Xeroderma pigmentosum, complementation group C (*XPC)* is located on 3p25.1 and encodes a protein involved in nucleotide excision repair. The identified mutation presumably truncates all functional domains of the XPC protein, which likely results in the loss of protein function.

**Conclusion:**

The study expands the knowledge of the mutational spectrum of *XPC* and is valuable for genetic counseling of affected individuals and their families.

## Graphical abstract

In this study, we report a novel protein truncating mutation (p.Asp98*) in *XPC* of xeroderma pigmentosum patient. The identified mutation presumably truncates all functional domains of the XPC protein, which likely results in the loss of protein function. The study expands the knowledge of the mutational spectrum of XPC and would be valuable for genetic counseling of affected individuals and their families.

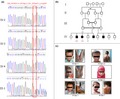

## 1 INTRODUCTION

Xeroderma pigmentosum (XP) is a rare genetic disorder caused by mutations in genes involved in the nucleotide excision repair (NER) pathway. The disease is characterized by severe sunlight sensitivity, changes in skin pigmentation, sunburn, and skin cancer (Jeppesen, Bohr, & Stevnsner, 2011; Lehmann, McGibbon, & Stefanini, 2011; Rao, 2007; Rass, Ahel, & West, 2007). It follows an autosomal recessive mode of inheritance affecting both males and females equally (Lehmann et al., 2011). The disease occurs in all ethnicities worldwide with an estimated incidence of 2.3 per million live births in Western Europe (Kleijer et al., 2008). Higher incidences have been reported for Japan (1:20,000), North Africa, Pakistan, and the Middle East, in which consanguineous marriages are more frequent (Hirai et al., 2006). XP group C is the most common form of XP in Europe and North Africa, accounting for about one third of all the forms (Soufir et al., 2010).

To date, eight genes have been identified to be associated with autosomal recessive XP (Fassihi et al., 2016), including *XPA* (Salob, Webb, & Atherton, 1992), *ERCC3* (XPB) (Lehmann, 1982), *XPC* (Li, Bales, Peterson, & Legerski, 1993), *ERCC2* (XPD) (Frederick, Amirkhan, Schultz, & Friedberg, 1994) *DDB2* (XPE) (Nichols, Ong & Linn, 1996), *ERCC4* (XPF) (Fujiwara et al., 1985), *ERCC5* (XPG) (Keijzer et al., 1979), and *POLH* (XPV) (Masutani et al., 1999). Physiologically, most of the reported XP genes are involved in UV‐induced nucleotide excision repair (NER). The proteins encoded by *XPC* and *DDB2* are required to recognize DNA photoproducts and to initiate the NER pathway. After binding to the nondamaged strand opposite of the lesion, recruitment of the TFIIH complex containing ERCC3 and ERCC2 is required to open the DNA structure and to verify the chemical modification of the photoproduct. Lesion recognition leads to the formation of the preincision complex including XPA and ERCC5. Recruitment of ERCC4 interacting with XPA leads to incision at 5´ of the damage site, repair initiation by polymerases and associated factors, and 3´ incision via ERCC5 (Schärer, 2013).

Depending on the XP group, patients either have a defective repair mechanism or lack the NER mechanism entirely (Stary & Sarasin, 2002). Severe and prolonged sunburn response is found in about 60% of XP patients. Development of lentigines in sun exposed areas is characteristic for this disease. In XP patients, incidence of nonmelanoma skin cancer is increased 10,000‐fold compared to melanoma skin cancer where the risk is 2,000‐fold higher under the age of 20 years (Bradford et al., 2011). In some patients, a varying degree of neurological and ocular symptoms, such as cognitive impairment, progressive hearing loss, acquired microcephaly, ataxia and visual impairment, can be observed (Lehmann et al., 2011; Andrews, Barrett, & Robbins, 1978; Stefanini & Kraemer, 2008).

To date, there is no cure for XP and, if untreated and without precaution or preventive strategies, will lead to premature death as a result of skin cancer, neurologic degeneration, or internal cancer (Kraemer & DiGiovanna, 2016).

## 2 MATERIALS AND METHODS

The current study was approved by the ethical review board of the Gomal University, D.I.Khan, Pakistan. Prior approval was obtained from guardians of volunteer participants for blood sampling, clinical and molecular characterization, and publication of the data. Herein the present study, we enrolled a consanguineous Pakistani family from a village nearby Bhakkar city in Punjab province, Pakistan. A pedigree was constructed and analyzed to elucidate the consanguineous relationship among the parents and to determine the mode of disease inheritance.

Clinical characterization was performed by documenting apparent features exhibited by the patients and laboratory tests including skin histopathology, complete blood count, urine analysis, as well as liver and renal function tests. Additionally, DNA was isolated from whole blood samples for molecular analysis via phenol‐chloroform extraction.

To elucidate the genetic cause of the disease, whole genome SNP genotyping was carried out through Illumina Infinium^®^ Global Screening Array‐24v1.0, and the data were analyzed using GenomeStudio 2.0 Software (Illumina) for homozygosity mapping. The subsequent mutation screening and segregation analysis was performed through Sanger DNA sequencing.

## 3 RESULTS

### 3.1 Clinical description

Clinical diagnosis of patients confirmed XP in all patients. The patients were presented with dense black spotting (lentigines) on sun exposed parts of the body (face, neck, and limbs), indicating extreme sensitivity to solar UV radiation. Abnormal dermal pigmentation in the form of macules was also observed on certain parts of the body. The patients displayed an extreme degree of photophobia, however, no ophthalmic lesions were detected and vision was normal in all patients. Blood analysis of a female patient with progressive health deterioration indicated a high white blood cell count and alkaline phosphatase levels. Histopathologic analysis of the patient's skin biopsy, taken from nose, revealed a necrotic tumor. Otherwise, blood biochemistry, liver and renal function tests as well as urine analysis reports were normal. A clinical summary of all XP patients is given in Table 1.

**Table 1:** Clinical features of patients suffering from Xeroderma Pigmentosum

Pedigree ID	IV‐3	IV‐2	IV‐5
Gross diagnosis	Xeroderma Pigmentosum	Xeroderma Pigmentosum	Xeroderma Pigmentosum
Age at last visit (during 2018)	11 years	13 years	7 years
Gender	Female	Male	Male
Age of disease onset	2–4 months	2–4 months	2–4 months
General physique	Slightly weak	Slightly weak	Slightly weak
Skin cancer	Not reported till last visit	Not reported till last visit	Not reported till last visit
Skin atrophy/lesions	Yes	Yes	Yes
Histopathology	Necrotic tumor	Not performed	Not performed
Muscle degeneration	No	No	No
Neurological symptoms	No	No	No
Wound healing	Delayed	Delayed	Delayed
Eye sight	Normal	Normal	Normal
Nystagmus	No	No	No
Color blindness	No	No	No
Strabismus	Yes	Yes	No
Deafness	No	No	No
Photophobia	Yes	Yes	Yes
Night blindness	No	No	No
Sweating	Normal	Normal	Normal
Hairs and nails	Normal	Normal	Normal

### 3.2 Genetic findings

**Figure 1:**
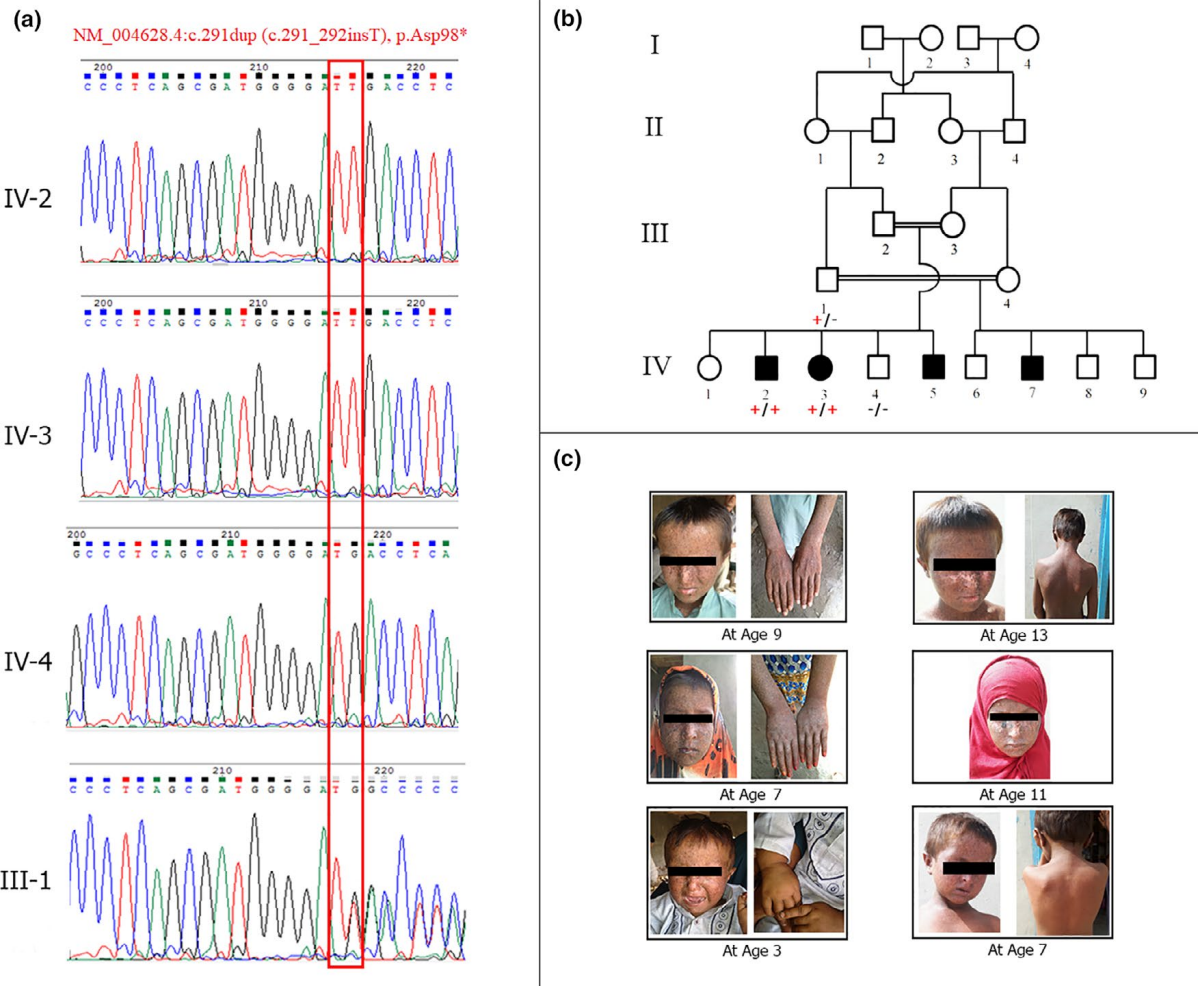
(a) Sequence chromatogram of affected patients (IV‐2 and IV‐3), unaffected sibling (IV‐4) and carrier father (III‐1). The site of mutation is framed in red. (b) The family tree comprises four generations with two consanguineous loops and four affected individuals in the last generation. The genotype status +/+ (homozygous insertion), +/− (carrier) or −/− (wild type) is represented beneath the symbol of each analyzed individual. (c) Photographs of patients, at current age and 4 years ago, who are exhibiting the apparent features of the disease

Genome‐wide scan through SNP genotyping determined five homozygous stretches of more than 20 Mb in size. A 10 Mb large homozygous region on chromosome 3 (rs411041 to rs2062572) harbored *XPC*, which is known to be associated with XP. Subsequent Sanger sequencing of *XPC* revealed a novel insertion mutation NM_004628.4:c.291dupT (c.291_292insT) in its second exon. The identified single base insertion presumably leads to a premature stop codon (TGA) at position 98 (p.Asp98*) and thus predictably results in a truncated protein. Variant genotyping in the whole family confirmed its segregation with the disease phenotype (Figure 1).

## 4 DISCUSSION

Xeroderma pigmentosum is a genetic disorder that is characterized by severe sensitivity to UV radiation, however, there are certain disorders that phenotypically resemble XP, such as UV‐sensitive syndrome (UVsS). UVsS is associated with dermal photosensitivity increased skin pigmentation, freckling, and dry skin on sun exposed areas. However, the patients have no increased risk of developing skin cancer. Genetically, UVsS is caused by mutations in UVSSA, ERCC6, and ERCC8. Proteins encoded by these genes are involved in transcription‐coupled nucleotide excision repair (Ijaz et al., 2019). However, one affected individual (IV‐3) of the current study had developed skin melanoma and photophobia in addition to the common phenotypic features of XP and UVsS. Thus, based on the clinical findings, our family was diagnosed with XP. The clinical diagnosis was confirmed with the identification of the novel mutation c.291dup, p.Asp98* in *XPC*. The *XPC* (XPC complex subunit, DNA damage recognition and repair factor, previously termed as XP, complementation group C) gene is located on chromosome 3 (3p25.1.). The largest transcript encodes the XPC protein comprising 940 amino acids, which is required for damage recognition in nucleotide excision repair (Schäfer et al., 2013). The full length protein contains eight domains, however, the novel nonsense mutation c.291dup, p.Asp98* presumably truncates all the functional domains of XPC, including multiple Rad4 beta‐hairpin domains, Transglutaminase‐like superfamily, Papain‐like cysteine peptidase superfamily, and DNA_repair_Rad4 domain. Hence, it is proposed that this mutation may lead to a loss of protein function. As a result, initiation of the NER pathway fails as DNA damage recognition does not take place.

According to HGMD^®^, most protein truncating mutations (loss‐of‐function mutation) lead to XP phenotype, whereas, missense variants are reported to be associated with cancer susceptibility. To date, at least 100 mutations have been identified in this gene, The majority of reported mutations in *XPC* are nonsense mutations or frameshift mutations, usually resulting in premature termination codons (HGMD® Professional 2019.1, accessed on 2019‐10‐07, Stenson et al., 2003). Both types either lead to an early termination of protein synthesis or to nonsense‐mediated mRNA decay in which the defective mRNA is prematurely degraded (Chavanne et al., 2000; Gozukara et al., 2001; Khan et al., 1998, 2006; Li et al., 1993; Ridley, Colley, Wynford‐Thomas, & Jones, 2005).

## 5 CONCLUSION

The present genetic study reports a novel frameshift mutation NM_004628.4:c291dup leading to a premature stop codon in *XPC*. The identification of this mutation further contributes to the genotype‐phenotype correlation of XPC‐associated XP. The study expands the knowledge of the mutational spectrum of XPC and is valuable for genetic counseling of affected individuals and their families.

## CONFLICT OF INTEREST

The authors declare that they have no financial as well as competing interest.

## AUTHORS CONTRIBUTION

*MZA, MAK, EU *and* SA* have recruited the patients, collected the samples, and performed clinical work. *JB, ES *and* EP* carried out molecular analysis. *MAK, CW, JB* and *MZA* remained involved in manuscript drafting. *MAK *and* CW* designed project, performed data analysis, and drafted the final version of manuscript. All authors have read and approved this final version.

## References

[mgg31060-bib-0001] Andrews, A. D. , Barrett, S. F. , & Robbins, J. H. (1978). Xeroderma pigmentosum neurological abnormalities correlate with colony‐forming ability after ultraviolet radiation. Proceedings of the National Academy of Sciences, 75(4), 1984–1988. 10.1073/pnas.75.4.1984 PMC392467 273925

[mgg31060-bib-0002] Bradford, P. T. , Goldstein, A. M. , Tamura, D. , Khan, S. G. , Ueda, T. , Boyle, J. , … Kraemer, K. H. (2011). Cancer and neurologic degeneration in xeroderma pigmentosum: Long term follow‐up characterises the role of DNA repair. Journal of Medical Genetics, 48(3), 168–176. 10.1136/jmg.2010.083022 21097776 PMC3235003

[mgg31060-bib-0003] Chavanne, F. , Broughton, B. C. , Pietra, D. , Nardo, T. , Browitt, A. , Lehmann, A. R. , & Stefanini, M. (2000). Mutations in the XPC gene in families with xeroderma pigmentosum and consequences at the cell, protein, and transcript levels. Cancer Research, 60(7), 1974–1982. 10766188

[mgg31060-bib-0004] Fassihi, H. , Sethi, M. , Fawcett, H. , Wing, J. , Chandler, N. , Mohammed, S. , … Lehmann, A. R. (2016). Deep phenotyping of 89 xeroderma pigmentosum patients reveals unexpected heterogeneity dependent on the precise molecular defect. Proceedings of the National Academy of Sciences, 113(9), E1236–E1245. 10.1073/pnas.1519444113 PMC4780618 26884178

[mgg31060-bib-0005] Frederick, G. D. , Amirkhan, R. H. , Schultz, R. A. , & Friedberg, E. C. (1994). Structural and mutational analysis of the xeroderma pigmentosum group D (XPD) gene. Human Molecular Genetics, 3(10), 1783–1788. 10.1093/hmg/3.10.1783 7849702

[mgg31060-bib-0006] Fujiwara, Y. , Ichihashi, M. , Uehara, Y. , Matsumoto, A. , Yamamoto, Y. , Kano, Y. , & Tanakura, Y. (1985). Xeroderma pigmentosum groups C and F: Additional assignments and a review of the subjects in Japan. Journal of Radiation Research, 26(4), 443–449. 10.1269/jrr.26.443. 3834095

[mgg31060-bib-0007] Gozukara, E. M. , Khan, S. G. , Metin, A. , Emmert, S. , Busch, D. B. , Shahlavi, T. , … Kraemer, K. H. (2001). A stop codon in xeroderma pigmentosum group C families in Turkey and Italy: Molecular genetic evidence for a common ancestor. Journal of Investigative Dermatology, 117(2), 197–204. 10.1046/j.1523-1747.2001.01424.x 11511294

[mgg31060-bib-0008] Hirai, Y. , Kodama, Y. , Moriwaki, S.‐I. , Noda, A. , Cullings, H. M. , MacPhee, D. G. , … Nakamura, N. (2006). Heterozygous individuals bearing a founder mutation in the XPA DNA repair gene comprise nearly 1% of the Japanese population. Mutation Research/Fundamental and Molecular Mechanisms of Mutagenesis, 601(1–2), 171–178. 10.1016/j.mrfmmm.2006.06.010 16905156

[mgg31060-bib-0009] Ijaz, A. , Wolf, S. , Mandukhail, S. R. , Basit, S. , Betz, R. C. , & Wali, A. (2019). UV‐sensitive syndrome: Whole exome sequencing identified a nonsense mutation in the gene UVSSA in two consanguineous pedigrees from Pakistan. Journal of Dermatological Science, 95(3), 113–118. 10.1016/j.jdermsci.2019.08.003 31421932

[mgg31060-bib-0010] Jeppesen, D. K. , Bohr, V. A. , & Stevnsner, T. (2011). DNA repair deficiency in neurodegeneration. Progress in Neurobiology, 94(2), 166–200. 10.1016/j.pneurobio.2011.04.013 21550379 PMC3123739

[mgg31060-bib-0011] Keijzer, W. , Jaspers, N. , Abrahams, P. J. , Taylor, A. , Arlett, C. F. , Zelle, B. , … Bootsma, D. (1979). A seventh complementation group in excision‐deficient xeroderma pigmentosum. Mutation Research/Fundamental and Molecular Mechanisms of Mutagenesis, 62(1), 183–190. 10.1016/0027-5107(79)90231-8 492197

[mgg31060-bib-0012] Khan, S. G. , Levy, H. L. , Legerski, R. , Quackenbush, E. , Reardon, J. T. , Emmert, S. , … Kraemer, K. H. (1998). Xeroderma pigmentosum group C splice mutation associated with autism and hypoglycinemia. Journal of Investigative Dermatology, 111(5), 791–796. 10.1046/j.1523-1747.1998.00391.x 9804340

[mgg31060-bib-0013] Khan, S. G. , Oh, K. S. , Shahlavi, T. , Ueda, T. , Busch, D. B. , Inui, H. , … DiGiovanna, J. J. (2006). Reduced XPC DNA repair gene mRNA levels in clinically normal parents of xeroderma pigmentosum patients. Carcinogenesis, 27(1), 84–94. 10.1093/carcin/bgi204 16081512

[mgg31060-bib-0014] Kleijer, W. J. , Laugel, V. , Berneburg, M. , Nardo, T. , Fawcett, H. , Gratchev, A. , … Lehmann, A. R. (2008). Incidence of DNA repair deficiency disorders in western Europe: Xeroderma pigmentosum, Cockayne syndrome and trichothiodystrophy. DNA Repair, 7(5), 744–750. 10.1016/j.dnarep.2008.01.014 18329345

[mgg31060-bib-0015] Kraemer, K. H. , & DiGiovanna, J. J. (2016). Xeroderma pigmentosum In Adam M. P., Ardinger H. H., Pagon R. A., Wallace S. E., Bean L. J. H., Stephens K. & Amemiya A., (Eds.), GeneReviews®[Internet]. Seattle: University of Washington. 20301571

[mgg31060-bib-0016] Lehmann, A. R. (1982). Three complementation groups in Cockayne syndrome. Mutation Research/Fundamental and Molecular Mechanisms of Mutagenesis, 106(2), 347–356. 10.1016/0027-5107(82)90115-4 6185841

[mgg31060-bib-0017] Lehmann, A. R. , McGibbon, D. , & Stefanini, M. (2011). Xeroderma pigmentosum. Orphanet Journal of Rare Diseases, 6(1), 70 10.1186/1750-1172-6-70 22044607 PMC3221642

[mgg31060-bib-0018] Li, L. , Bales, E. S. , Peterson, C. A. , & Legerski, R. J. (1993). Characterization of molecular defects in xeroderma pigmentosum group C. Nature Genetics, 5(4), 413 10.1038/ng1293-413 8298653

[mgg31060-bib-0019] Masutani, C. , Kusumoto, R. , Yamada, A. , Dohmae, N. , Yokoi, M. , Yuasa, M. , … Hanaoka, F. (1999). The XPV (xeroderma pigmentosum variant) gene encodes human DNA polymerase η. Nature, 399(6737), 700 10.1038/21447 10385124

[mgg31060-bib-0020] Nichols, A. F. , Ong, P. , & Linn, S. (1996). Mutations specific to the xeroderma pigmentosum group E Ddb phenotype. Journal of Biological Chemistry, 271(40), 24317–24320. 10.1074/jbc.271.40.24317 8798680

[mgg31060-bib-0021] Rao, K. S. (2007). Mechanisms of disease: DNA repair defects and neurological disease. Nature Reviews Neurology, 3(3), 162–172. 10.1038/ncpneuro0448 17342192

[mgg31060-bib-0022] Rass, U. , Ahel, I. , & West, S. C. (2007). Defective DNA repair and neurodegenerative disease. Cell, 130(6), 991–1004. 10.1016/j.cell.2007.08.043 17889645

[mgg31060-bib-0023] Ridley, A. J. , Colley, J. , Wynford‐Thomas, D. , & Jones, C. J. (2005). Characterisation of novel mutations in Cockayne syndrome type A and xeroderma pigmentosum group C subjects. Journal of Human Genetics, 50(3), 151 10.1007/s10038-004-0228-2 15744458

[mgg31060-bib-0024] Salob, S. P. , Webb, D. K. H. , & Atherton, D. J. (1992). A child with xeroderma pigmentosum and bone marrow failure. British Journal of Dermatology, 126(4), 372–374. 10.1111/j.1365-2133.1992.tb00681.x 1571258

[mgg31060-bib-0025] Schäfer, A. , Hofmann, L. , Gratchev, A. , Laspe, P. , Schubert, S. , Schürer, A. , … Emmert, S. (2013). Molecular genetic analysis of 16 XP‐C patients from Germany: Environmental factors predominately contribute to phenotype variations. Experimental Dermatology, 22(1), 24–29. 10.1111/exd.12052 23173980

[mgg31060-bib-0026] Schärer, O. D. (2013). Nucleotide excision repair in eukaryotes. Cold Spring Harbor Perspectives in Biology, 5(10), a012609 10.1101/cshperspect.a012609 24086042 PMC3783044

[mgg31060-bib-0027] Soufir, N. , Ged, C. , Bourillon, A. , Austerlitz, F. , Chemin, C. , Stary, A. , … Sarasin, A. (2010). A prevalent mutation with founder effect in xeroderma pigmentosum group C from North Africa. Journal of Investigative Dermatology, 130(6), 1537–1542. 10.1038/jid.2009.409 20054342

[mgg31060-bib-0028] Stary, A. , & Sarasin, A. (2002). The genetics of the hereditary xeroderma pigmentosum syndrome. Biochimie, 84(1), 49–60. 10.1016/S0300-9084(01)01358-X 11900876

[mgg31060-bib-0029] Stefanini, M. , & Kraemer, K. H. (2008). Xeroderma pigmentosum In Ruggieri M., Pascual‐Castroviejo I., & Di Rocco C. (Eds.), Neurocutaneous disorders phakomatoses and hamartoneoplastic syndromes. Vienna: Springer.

[mgg31060-bib-0030] Stenson, P. D. , Ball, E. V. , Mort, M. , Phillips, A. D. , Shiel, J. A. , Thomas, N. S. , … Cooper, D. N. (2003). Human Gene Mutation Database (HGMD): 2003 update. Human Mutation, 21(6), 577–581. 10.1002/humu.10212 12754702

